# Development of Osteoarthritis in Adults With Type 2 Diabetes Treated With Metformin vs a Sulfonylurea

**DOI:** 10.1001/jamanetworkopen.2023.3646

**Published:** 2023-03-20

**Authors:** Matthew C. Baker, Khushboo Sheth, Yuhan Liu, Di Lu, Rong Lu, William H. Robinson

**Affiliations:** 1 Division of Immunology and Rheumatology, Department of Medicine, Stanford University, Stanford, California; 2 Chinook Therapeutics, Seattle, Washington; 3 The VA Palo Alto Health Care System, Palo Alto, California; 4 The Quantitative Sciences Unit, Division of Biomedical Informatics Research, Department of Medicine, Stanford University, Stanford, California

## Abstract

**Importance:**

Metformin may have a protective association against developing osteoarthritis (OA), but robust epidemiological data are lacking.

**Objective:**

To determine the risk of OA and joint replacement in individuals with type 2 diabetes treated with metformin compared with a sulfonylurea.

**Design, Setting, and Participants:**

This retrospective cohort study used claims data from the Optum deidentified Clinformatics Data Mart Database between December 2003 and December 2019. Participants included individuals aged 40 years or older with at least 1 year of continuous enrollment and type 2 diabetes. Individuals with type 1 diabetes or a prior diagnosis of OA, inflammatory arthritis, or joint replacement were excluded. Time-conditional propensity score matching was conducted using age, sex, race, Charlson comorbidity score, and treatment duration to create a prevalent new-user cohort. Data were analyzed from April to December 2021.

**Exposures:**

Treatment with metformin or a sulfonylurea.

**Main Outcomes and Measures:**

The outcomes of interest were incident OA and joint replacement. Cox proportional hazard models were used to calculate adjusted hazard ratios (aHRs) of incident OA and joint replacement. In a sensitivity analysis, individuals only ever treated with metformin were compared with individuals only ever treated with a sulfonylurea, allowing for longer-term follow up of the outcome (even after stopping the medication of interest).

**Results:**

After time-conditional propensity score matching, the metformin and control groups each included 20 937 individuals (mean [SD] age 62.0 [11.5] years; 24 379 [58.2%] males). In the adjusted analysis, the risk of developing OA was reduced by 24% for individuals treated with metformin compared with a sulfonylurea (aHR, 0.76; 95% CI, 0.68-0.85; *P* < .001), but there was no significant difference for risk of joint replacement (aHR, 0.80; 95% CI, 0.50-1.27; *P* = .34). In the sensitivity analysis, the risk of developing OA remained lower in individuals treated with metformin compared with a sulfonylurea (aHR, 0.77; 95% CI, 0.65-0.90; *P* < .001) and the risk of joint replacement remained not statistically significant (aHR, 1.04; 95% CI, 0.60-1.82; *P* = .89).

**Conclusions and Relevance:**

In this cohort study of individuals with diabetes, metformin treatment was associated with a significant reduction in the risk of developing OA compared with sulfonylurea treatment. These results further support preclinical and observational data that suggest metformin may have a protective association against the development of OA; future interventional studies with metformin for the treatment or prevention of OA should be considered.

## Key Points

**Question:**

Is metformin use associated with incidence of osteoarthritis (OA)?

**Findings:**

In this cohort study including 41 874 time-conditional propensity score–matched patients using metformin or a sulfonylurea, those treated with metformin had a lower estimated risk of developing OA.

**Meaning:**

These findings suggest that metformin use was associated with a lower incidence of OA, and future interventional studies with metformin for preventing OA could be considered.

## Teaser

This cohort study assesses the risk of osteoarthritis and joint replacement in individuals with type 2 diabetes treated with metformin vs a sulfonylurea.

## Introduction

Osteoarthritis (OA) is the most common form of arthritis, affecting more than 32.5 million individuals in the United States, and it is one of the major contributors to global years lived with disability.^1,2,3^ Current therapeutic strategies for OA are focused on symptomatic management, and there are no effective disease-modifying treatments to halt, slow, or reverse the progression of OA.^4^ This represents a large unmet need.

Metformin is a biguanide derivative that is used as first-line treatment of type 2 diabetes by inhibiting hepatic gluconeogenesis and increasing muscle insulin sensitivity.^5^ Metformin is generally considered safe in most patient populations and is available at a low cost.^6^ In addition to its primary role in the treatment of diabetes, metformin has been purported to have anti-inflammatory, antiaging, anticancer, pro–weight loss, and immunomodulatory effects.^7^

Emerging evidence suggests that metformin may be useful for the treatment or prevention of OA.^8,9,10,11,12,13,14,15^ Preclinical studies suggest that metformin has disease-modifying properties in OA models in mice, rats, and macaque monkeys.^9,10^ Observational studies in humans have also largely supported the use of metformin associated with preventing the development of OA or the need for joint replacement.^8,13,14,15^ However, these studies have predominately focused on progression of preexisting OA (as opposed to the development of incident OA); many have not accounted for concomitant antidiabetic medication use, thus failing to fully isolate the effects of metformin; and some have suffered from immortal time bias related to the comparison of metformin users and nonusers (as opposed to an active treatment control arm).

Based on the available preclinical and observational human data, metformin use may prevent the development of OA. Therefore, we conducted a large, nationwide cohort study using time-conditional propensity score matching to evaluate the risk of developing OA and the need for joint replacement in individuals with diabetes who were treated with metformin compared with a sulfonylurea.

## Methods

This cohort study was deemed exempt from review and informed consent by the Stanford University institutional review board because data were deidentified. This study adhered to the Strengthening the Reporting of Observational Studies in Epidemiology (STROBE) reporting guideline for cohort studies.

### Study Design and Data Source

This was a retrospective cohort study using data from the Optum Clinformatics Data Mart Database (CDM), a deidentified database derived from a large, adjudicated claims data warehouse, from December 1, 2003, to December 31, 2019. This data set includes more than 15 million individuals annually from across the United States who are privately insured or Medicare Advantage Part D members. This provides a geographically representative sample; however, it does not include recipients of Medicaid, and thus the resulting study population has a higher socioeconomic status than the total population with diabetes at risk for OA.

### Study Population

We included individuals aged 40 years or older with at least 1 year of continuous enrollment in the Optum CDM database before the first *International Classification of Diseases, Ninth Revision (ICD-9*) (before October 1, 2015) or *International Statistical Classification of Diseases and Related Health Problems, Tenth Revision (ICD-10)* (after October 1, 2015) diagnosis of diabetes and first prescription for either metformin or a sulfonylurea. The index date for both groups was the first fill date for the drug of interest. Individuals with diabetes were defined as having at least 2 *ICD-9* or *ICD-10* codes for type 2 diabetes separated by 14 days or more (eTable 1 in Supplement 1).^16,17^ We excluded individuals with type 1 diabetes, patients with the first diagnosis of diabetes occurring after the start date of metformin or the sulfonylurea, patients started on metformin and a sulfonylurea at the same time, and patients using combination metformin or sulfonylurea medications. Individuals with prior diagnoses of OA or any inflammatory arthritis or with joint replacement based on *Current Procedural Terminology* (*CPT*) codes prior to the index date or within the first 90 days of the index date were also excluded (eTable 1 in Supplement 1). The lookback period for these exclusion criteria included all available data.

### Exposure

The exposed group included individuals with diabetes treated with metformin for at least 90 days, and the control group included individuals with diabetes who were treated with a sulfonylurea medication for at least 90 days. Those who were initially treated with metformin and then switched to a sulfonylurea could contribute data to both groups. People who switched to metformin after being treated with a sulfonylurea contributed data to the sulfonylurea group and were then censored when switching. This was done to maximize the number of individuals contributing data to the sulfonylurea group, which was substantially smaller in size compared with the metformin group.

### Outcome Ascertainment

The primary end point was the time to diagnosis of incident OA starting 90 days after the index date. Ninety days was chosen as the minimum period of time in which a treatment effect was likely to be observed. OA was defined as 2 or more *ICD-9* or *ICD-10* codes for OA separated by 14 days or more.^18^ The secondary end point was the time to joint replacement starting 90 days after the index date. Joint replacement was defined as a documented *CPT* code for hip or knee joint replacement. Individuals were followed from 90 days after the index date until they received a diagnosis of OA, underwent knee or hip joint arthroplasty, stopped treatment with metformin or a sulfonylurea (defined as the start of a gap of 90 days or more with no treatment), received any diabetes treatment other than metformin or a sulfonylurea, were no longer present in the Optum CDM database, or until the end of the follow-up period (December 31, 2019) (eFigure 1 in Supplement 1).

### Statistical Analysis

Given that sulfonylureas are often used as second-line agents after metformin, there may be inherent biases in the comparison of individuals treated with metformin and individuals treated with a sulfonylurea. For this reason, we compared these 2 groups using a prevalent new-user cohort design (eFigure 2 in Supplement 1).^19^ This allowed us to compare the first-line treatment (metformin) with the second-line treatment (sulfonylurea) using time-based exposure sets to identify matched individuals at the same point in the course of disease, thus helping to eliminate potential time-lagging bias.^20^ The cohort included all individuals treated with a sulfonylurea. For each person treated with a sulfonylurea, a matched person treated with metformin was identified based on time-based exposure sets defined as time intervals (±15 days) from the first prescription of metformin to the first dose of sulfonylurea. Individuals were matched 1:1 on time-conditional propensity scores using conditional logistic regression adjusting for age, sex, race and ethnicity (reported in the database as Asian, Black, Hispanic, White, or unknown), Charlson comorbidity score, and treatment duration to estimate the propensity to receive a sulfonylurea. Race and ethnicity were included in analyses because racial differences in OA prevalence and severity may exist.^21^ For time-conditional propensity score matching, we started chronologically with the first individual prescribed a sulfonylurea and selected the individual from the exposure set with the closest time-conditional propensity score. Once a person had been selected into the comparator group, they were no longer considered in subsequent exposure sets as potential comparators.

Baseline characteristics of individuals in both groups after time-conditional propensity score matching were compared. We used the Quan-Deyo method to calculate the Charlson comorbidity score.^22^ Standardized mean differences (SMDs) were calculated using the tableone package in R software version 4.1.1 (R Project for Statistical Computing). Missing data for the categorical variables are reported as unknown. For the continuous variables, there were no missing data. Incidence rates (IRs) and 95% Wald CIs were calculated for developing OA and undergoing joint replacement, and Cox proportional hazard models were used to assess the hazard ratio (HR) and 95% CI of developing OA and joint replacement among individuals with diabetes treated with metformin compared with a sulfonylurea after adjusting for age, sex, race and ethnicity, geographical region, education, Charlson comorbidity score, and outpatient visit frequency. IRs were reported as the number of events per 1000 person-years. Kaplan-Meier curves were created to report the probability of developing OA over a certain time interval. A stratified analysis was conducted using the matched data to evaluate the treatment outcomes of metformin compared with a sulfonylurea. The results were stratified by the matched pairs for individuals treated with a sulfonylurea with prior metformin exposure and those without prior metformin exposure.

All statistical analyses were conducted using SAS version 9.4 (SAS Institute) and R version 4.1.1. Charlson comorbidity scores were calculated using the icd package.^23^ All 95% CIs and *P* values were based on 2-sided hypothesis tests, where *P* < .05 was considered statistically significant. The statistical analysis plan is presented in the eAppendix in Supplement 1. Data were analyzed from April to December 2021.

The robustness of our results was examined through a sensitivity analysis comparing individuals treated with metformin with individuals treated with a sulfonylurea who were only ever treated with those medications (eFigure 1 in Supplement 1). This allowed for longer-term follow-up of the outcome even after people had stopped the medication of interest, since with this analysis, no new medications could subsequently be introduced that could influence the outcome of interest. For both groups, the index date was the first fill date for the drug of interest. For the exposed group, we included individuals with diabetes who were ever treated with metformin and never treated with any additional diabetes medications during their entire follow-up period. For the control group, we included individuals with diabetes who were ever treated with a sulfonylurea medication and never treated with any additional diabetes medications during their entire follow-up period. We excluded people who ever received any diabetes medication other than metformin or a sulfonylurea (including combination metformin or sulfonylurea medications). We conducted 1:1 propensity score matching of individuals in the exposed group and individuals in the control group using the same variables as in the primary analysis. We used a caliper of width equal to 0.2 of SD of the logit of the propensity score.^24,25^

## Results

### Patient Characteristics

After time-conditional propensity score matching, 20 937 individuals were included in each group (mean [SD] age 62.0 [11.5] years; 24 379 [58.2%] males; mean [SD] Charlson comorbidity score 0.71 [1.35]) (eFigure 3 in Supplement 1; Table 1). In the metformin group, the mean (SD) duration of treatment was 12.34 (10.70) months, compared with 12.56 (12.37) months in the sulfonylurea group (Table 1).

**Table 1.** Patient Characteristics of Patients With Diabetes Who Used Metformin and Time-Conditional Propensity Score-Matched Patients Who Used a Sulfonylurea at Cohort Entry

Characteristic	Patients, No. (%)			SMD
	Total cohort (N = 41 874)	Metformin (n = 20 937)	Sulfonylurea (n = 20 937)	
Age, mean (SD), y	62.0 (11.5)	62.0 (11.1)	62.1 (11.9)	0.013
Sex				
Female	17 495 (41.8)	8747 (41.8)	8748 (41.8)	<0.001
Male	24 379 (58.2)	12 190 (58.2)	12 189 (58.2)	
Race and ethnicity				
Asian	1837 (4.4)	932 (4.5)	905 (4.3)	0.017
Black	5797 (13.8)	2860 (13.7)	2937 (14.0)	
Hispanic	5507 (13.2)	2798 (13.4)	2709 (12.9)	
White	28 733 (68.6)	14 347 (68.5)	14 386 (68.7)	
Region				
North Central	9599 (22.9)	4635 (22.1)	4964 (23.7)	0.072
Northeast	3592 (8.6)	1872 (8.9)	1720 (8.2)	
South	19 196 (45.8)	9431 (45.0)	9765 (46.6)	
West	9434 (22.5)	4978 (23.8)	4456 (21.3)	
Unknown	53 (0.1)	21 (0.1)	32 (0.2)	
Education				
<12th Grade	376 (0.9)	165 (0.8)	211 (1.0)	0.140
High school diploma	14 184 (33.9)	6528 (31.2)	7656 (36.6)	
<Bachelor’s degree	22 320 (53.3)	11 406 (54.5)	10 914 (52.1)	
≥Bachelor’s degree	4814 (11.5)	2748 (13.1)	2066 (9.9)	
Unknown	180 (0.4)	90 (0.4)	90 (0.4)	
Charlson comorbidity score				
0	29 048 (69.4)	14 493 (69.2)	14 555 (69.5)	0.029
1-2	10 017 (23.9)	5008 (23.9)	5009 (23.9)	
3-4	2044 (4.9)	1017 (4.9)	1027 (4.9)	
5-6	380 (0.9)	217 (1.0)	163 (0.8)	
>6	385 (0.9)	202 (1.0)	183 (0.9)	
Charlson comorbidity score, mean (SD)	0.71 (1.35)	0.69 (1.35)	0.74 (1.35)	0.038
Outpatient annual visit frequency, mean (SD)	6.98 (7.08)	6.53 (6.32)	7.43 (7.75)	0.127
Treatment duration, mean (SD), mo	12.45 (11.56)	12.34 (10.70)	12.56 (12.37)	0.019
Follow-up time, mean (SD), mo	9.45 (11.56)	9.34 (10.70)	9.56 (12.37)	0.019

Abbreviation: SMD, standardized mean difference.

### Primary Outcome

Using a prevalent new-user cohort design and after time-conditional propensity score matching, the IR of OA for individuals treated with metformin was 27.5 events per 1000 person-years, compared with 39.6 events per 1000 person-years for individuals treated with a sulfonylurea (Table 2). After adjusting for age, sex, race and ethnicity, geographical region, education, Charlson comorbidity score, and outpatient visit frequency, individuals who received metformin were 24% less likely to develop OA compared with those who were treated with a sulfonylurea (aHR, 0.76; 95% CI, 0.68-0.85; *P* < .001) (Table 2).

**Table 2.** Incidence and Risk of Developing OA and Undergoing Joint Replacement in Patients Treated With Metformin vs a Sulfonylurea

Measure	Metformin (n = 20 937)	Sulfonylurea (n = 20 937)
**Incident OA**		
Events, No. (%)	568 (2.7)	817 (3.9)
Person-years, No.	20 653	20 644
IR (95% CI), per 1000 person-years	27.5 (25.3-29.8)	39.6 (36.9-42.4)
IRR (95% CI)	0.69 (0.62-0.77)	1 [Reference]
HR (95% CI)		
Crude	0.69 (0.62-0.77)	1 [Reference]
Adjusted^a^	0.76 (0.68-0.85)	1 [Reference]
**Joint replacement**		
Events, No. (%)	31 (0.15)	45 (0.21)
Person-years, No.	21 202	21 553
IR (95% CI), per 1000 person-years	1.5 (1.0-2.1)	2.1 (1.5-2.8)
IRR (95% CI)	0.70 (0.44-1.11)	1 [Reference]
HR (95% CI)		
Crude	0.72 (0.46-1.14)	1 [Reference]
Adjusted^a^	0.80 (0.50-1.27)	1 [Reference]

Abbreviations: HR, hazard ratio; IR, incidence rate; IRR, incidence rate ratio; OA, osteoarthritis.

^a^ Adjusted for age, sex, race and ethnicity, geographical region, education, Charlson comorbidity score, and outpatient visit frequency.

In the stratified analysis, the risk of developing OA in people treated with metformin compared with those treated with a sulfonylurea with prior metformin use was no longer statistically significant (aHR, 0.92; 95% CI, 0.76-1.12), but the risk of developing OA in individuals treated with metformin compared with individuals treated with a sulfonylurea with no prior metformin use was still significantly lower (aHR, 0.71; 95% CI, 0.62-0.81) (eTable 2 in Supplement 1). The Kaplan-Meier plot demonstrated that individuals who received metformin were less likely to be diagnosed with OA over time, with a visual separation of the curves by 1 year from the index date, and this persisted during the follow-up period of more than 6 years (Figure).

**Figure.**
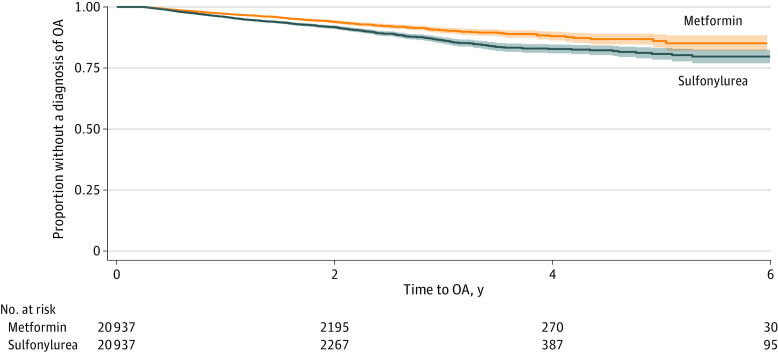
Kaplan-Meier Curve of Time to Osteoarthritis (OA) Diagnosis in Patients Treated With Metformin Compared With Those Treated With Sulfonylurea After Time-Conditional Propensity Score Matching

### Secondary Outcome

In the prevalent new-user cohort analysis, the IR of joint replacement for individuals treated with metformin was 1.5 events per 1000 person-years, compared with 2.1 events per 1000 person-years for individuals treated with a sulfonylurea. There was no statistically significant reduction in the risk of undergoing joint replacement in people treated with metformin vs a sulfonylurea (aHR, 0.80; 95% CI, 0.50-1.27; *P* = .34) (Table 2).

### Sensitivity Analysis

In the sensitivity analysis, a total of 104 471 individuals were treated with metformin only, and 8277 individuals were treated with a sulfonylurea medication only (eFigure 4 in Supplement 1). After propensity score matching, 8277 people remained in each group; both groups had a similar mean (SD) age (metformin: 66.1 [11.9] years; sulfonylurea: 66.3 [12.5] years) and Charlson comorbidity score (metformin: 1.3 [2.0]; sulfonylurea: 1.3 [2.1]), and a total of 10 325 (62.4%) males (Table 3). The mean (SD) treatment duration for the metformin group was 18.4 (24.0) months compared with 19.8 (26.1) months for the sulfonylurea group (Table 3). Neither group received any additional diabetes medication after treatment with either metformin or a sulfonylurea ended. In the sensitivity analysis, after propensity score matching, the IR of OA for individuals who received metformin was 25.4 events per 1000 person-years, compared with 31.1 events per 1000 person-years for individuals treated with a sulfonylurea medication (Table 4). There was a 23% reduction in risk for the development of OA in people treated with metformin compared with patients treated with a sulfonylurea (aHR, 0.77; 95% CI, 0.65-0.90; *P* < .001) (Table 4). The protective association of metformin treatment compared with sulfonylurea treatment over time is visually represented in the Kaplan-Meier plot in eFigure 5 in Supplement 1. No difference in joint replacement was seen between groups.

**Table 3.** Patient Characteristics in the Sensitivity Analysis Before and After 1:1 PS Matching

Characteristic	Before PS matching			After PS matching		
	Patients, No. (%)		SMD	Patients, No. (%)		SMD
	Metformin (n = 104 471)	Sulfonylurea (n = 8277)		Metformin (n = 8277)	Sulfonylurea (n = 8277)	
Age, mean (SD), y	59.4 (11.1)	66.3 (12.5)	0.584	66.1 (11.9)	66.3 (12.5)	0.019
Sex						
Female	45 151 (43.2)	3144 (38.0)	0.107	3085 (37.3)	3144 (38.0)	0.015
Male	59 320 (56.8)	5133 (62.0)		5192 (62.7)	5133 (62.0)	
Race and ethnicity						
Asian	5713 (5.5)	356 (4.3)	0.123	353 (4.3)	356 (4.3)	0.029
Black	11 719 (11.2)	1054 (12.7)		988 (11.9)	1054 (12.7)	
Hispanic	15 160 (14.5)	972 (11.7)		1009 (12.2)	972 (11.7)	
White	63 766 (61.0)	5088 (61.5)		5142 (62.1)	5088 (61.5)	
Unknown	8113 (7.8)	807 (9.7)		785 (9.5)	807 (9.7)	
Education						
<12th Grade	877 (0.8)	103 (1.2)	0.194	74 (0.9)	103 (1.2)	0.162
High school diploma	30 030 (28.7)	2796 (33.8)		2341 (28.3)	2796 (33.8)	
<Bachelor’s degree	53 750 (51.4)	3992 (48.2)		4255 (51.4)	3992 (48.2)	
≥Bachelor’s degree	13 875 (13.3)	729 (8.8)		1024 (12.4)	729 (8.8)	
Unknown	5939 (5.7)	657 (7.9)		583 (7.0)	657 (7.9)	
Charlson comorbidity score						
0	70 374 (67.4)	4285 (51.8)	0.424	4227 (51.1)	4285 (51.8)	0.018
1-2	27 082 (25.9)	2383 (28.8)		2428 (29.3)	2383 (28.8)	
3-4	5070 (4.9)	985 (11.9)		1012 (12.2)	985 (11.9)	
5-6	1003 (1.0)	366 (4.4)		356 (4.3)	366 (4.4)	
>6	942 (0.9)	258 (3.1)		254 (3.1)	258 (3.1)	
Charlson comorbidity score, mean (SD)	0.6 (1.3)	1.3 (2.1)	0.413	1.3 (2.0)	1.3 (2.1)	0.018
Treatment duration, mean (SD), mo	24.4 (28.1)	19.8 (26.1)	0.169	18.4 (24.0)	19.8 (26.1)	0.058
Follow-up time, mean (SD), mo	31.9 (33.1)	32.4 (36.6)	0.013	28.8 (31.7)	32.4 (36.6)	0.104

Abbreviations: PS, propensity score; SMD, standardized mean difference.

**Table 4.** Incidence and Risk of Developing OA and Undergoing Joint Replacement in the Sensitivity Analysis of Patients Treated With Metformin vs a Sulfonylurea

Measure	Before PS-matching		After PS-matching	
	Metformin (n = 104 471)	Sulfonylurea (n = 8277)	Metformin (n = 8277)	Sulfonylurea (n = 8277)
**Incident OA**				
Events, No. (%)	7154 (6.9)	703 (8.5)	530 (6.4)	703 (8.5)
Person-years	288 795	22 588	20 890	22 588
IR (95% CI),per 1000 person-years	24.8 (24.2-25.4)	31.1 (28.8-33.4)	25.4 (23.3-27.5)	31.1 (28.8-33.4)
IRR (95% CI)	0.80 (0.77-0.83)	1 [Reference]	0.82 (0.77-0.87)	1 [Reference]
Adjusted HR (95% CI)^a^	0.79 (0.73-0.85)	1 [Reference]	0.77 (0.65-0.90)	1 [Reference]
**Joint replacement**				
Events, No. (%)	782 (0.8)	64 (0.8)	63 (0.8)	64 (0.8)
Person-years	309 650	25 040	22 384	25 040
IR (95% CI), per 1000 person-years	2.5 (2.3-2.7)	2.6 (2.0-3.2)	2.8 (2.1-3.5)	2.6 (2.0-3.2)
IRR (95% CI)	0.96 (0.84-1.10)	1 [Reference]	1.08 (0.90-1.29)	1 [Reference]
Adjusted HR (95% CI)^a^	1.04 (0.80-1.34)	1 [Reference]	1.04 (0.60-1.82)	1 [Reference]

Abbreviations: HR, hazard ratio; IR, incidence rate; IRR, incidence rate ratio; OA, osteoarthritis; PS, propensity score.

^a^ Adjusted for age, sex, race and ethnicity, Charlson comorbidity score, and treatment duration.

## Discussion

In this large retrospective cohort study, we found a 24% reduction in the risk of developing OA in individuals with diabetes treated with metformin compared with time-conditional propensity score–matched individuals treated with a sulfonylurea. When stratified by prior exposure to metformin within the sulfonylurea group, the observed benefit associated with metformin compared with sulfonylurea was attenuated in the people treated with a sulfonylurea with prior exposure to metformin compared with those treated with a sulfonylurea with no prior exposure to metformin. One possible hypothesis for this finding is that individuals in the sulfonylurea group with prior exposure to metformin derived a degree of long-lasting protection associated with the metformin exposure. In a sensitivity analysis comparing individuals only ever treated with metformin with individuals only ever treated with a sulfonylurea, allowing for longer-term follow-up of the outcome (even after stopping the medication of interest), we found a similar 23% reduction in the risk of developing OA in individuals treated with metformin.

This study supports prior literature demonstrating benefit in OA associated with treatment with metformin.^9,11,12^ Several preclinical studies have suggested a protective association of metformin in OA through activating AMP-activated protein kinase signaling, decreasing the level of matrix metalloproteinase 13, increasing autophagy and reducing chondrocyte apoptosis, and augmenting chondroprotective and anti-inflammatory properties of mesenchymal stem cells.^9,10,11,12^

Human data also support the use of metformin for the treatment or prevention of OA. In an observational study,^8^ individuals with obesity and knee OA who were treated with metformin were found to have a lower rate of medial cartilage volume loss compared with individuals not treated with metformin. A population-based cohort study reported a reduced incidence of total knee arthroplasty in individuals with preexisting OA and diabetes who had received a combination of metformin and a cyclooxygenase-2 inhibitor compared with a cyclooxygenase-2 inhibitor alone.^13^ Additional cohort studies have found that individuals with diabetes treated with metformin had a significantly reduced risk of total knee arthroplasty.^14,15^ One cohort study found no association between metformin use and incidence of developing OA; however, a systematic review of 10 preclinical and 5 human studies of OA concluded that metformin had chondroprotective, immunomodulatory, and analgesic associations.^26,27^ Our study provides further, robust epidemiological evidence that metformin may be associated with protection in the development and progression of OA in individuals with type 2 diabetes.

### Strengths and Limitations

Our study has several strengths. We used a large claims database covering individuals in a wide geographic area in the United States. We were able to exclude people with diabetes who were using additional treatments, thus reducing potential confounding from these medications and more effectively isolating the outcomes associated with metformin. We conducted our analysis using a prevalent new-user cohort design with time-conditional propensity score matching, which allowed us to compare persons using metformin users or sulfonylurea at the starting point of each medication, helping to avoid immortal time bias and time-lagging bias. We specifically selected individuals with type 2 diabetes who either required treatment with metformin alone or a sulfonylurea alone to create similar cohorts of people with mild diabetes. We were able to follow up individuals for up to 10 years to ascertain the outcome. We were also able to conduct a sensitivity analysis comparing individuals only ever treated with metformin with those only ever treated with a sulfonylurea, allowing for longer-term follow up for the outcome, which demonstrated similar results as our primary analysis.

Our study has several limitations. First, as this is a retrospective study using claims data, there may be residual or unmeasured confounders. To balance the covariates, we used propensity score matching and adjusted for important covariables. Second, we did not have data on body mass index, which is associated with OA. It is possible that metformin use resulted in more weight loss than sulfonylurea use, and the reduction in OA we observed was mediated primarily by weight loss. However, studies have shown that weight loss induced by metformin is modest, and a prior randomized clinical trial of diet and exercise that resulted in a similar degree of weight loss did not significantly reduce the risk of developing OA.^28,29,30^ We believe metformin likely exerts protective associations beyond what can be attributed to weight loss alone. Third, we also lacked data on level of physical activity or history of trauma to the involved joints, both of which can be associated with OA. However, these factors should not have affected whether patients received metformin or a sulfonylurea for the treatment of diabetes and are thus likely nondifferential between groups. Fourth, our study only evaluates the association of metformin with the development of OA in patients with diabetes, thus limiting its generalizability. Given the underlying metabolic derangements in patients with diabetes, it is possible that the benefits we observed from metformin treatment would not be seen in patients without diabetes. Fifth, the Optum CDM data set is limited to individuals with commercial or Medicare Advantage coverage, and therefore may not be representative of the entire US population. Sixth, we included people who switched from metformin to sulfonylureas, but not vice versa, to maximize the number of individuals contributing data to the sulfonylurea group, which was substantially smaller in size than the metformin group. This may have created some form of bias; however, we wanted to isolate the associations of metformin alone without potential confounding by prior treatment with a sulfonylurea. Seventh, we used *ICD-9*, *ICD-10*, and *CPT* codes for identification of diseases and outcomes, which could have led to misclassification of variables and outcomes; however, we believe this is likely to be nondifferential between groups. Eighth, we could not determine the degree of medication adherence in the any of the treatment groups.

## Conclusions

In our large, nationwide cohort study of individuals with diabetes, metformin treatment was associated with a significant reduction in the risk of developing OA compared with sulfonylurea treatment. Results from this study must be interpreted with caution due to the lack of data on body mass index, and the possibility that weight loss induced by metformin may have accounted for some of the benefit seen. Despite this limitation, this study further supports the preclinical and observational data that show metformin may have a protective association against the development of OA. Future interventional studies with metformin for the treatment or prevention of OA should be considered.

## Notes

Supplement 1. **eTable 1.** Codes Used for Patient Inclusion and Exclusion and Outcome Ascertainment **eTable 2.** Hazard Ratios of Developing Osteoarthritis and Undergoing Joint Replacement in Patients Treated With Metformin vs a Sulfonylurea, Stratified by Prior Metformin Use in the Sulfonylurea Group **eFigure 1.** Primary Analysis and Sensitivity Analysis Study Design **eFigure 2.** Prevalent New-User Study Design With Time-Based Exposure Sets Defined by a 15-Day Interval Surrounding the Timing of Sulfonylurea Initiation **eFigure 3.** Flowchart of Cohort Selection for Primary Analysis **eFigure 4.** Flowchart of Cohort Selection for Sensitivity Analysis **eFigure 5.** Kaplan-Meier Curve of Time to Osteoarthritis Diagnosis in the Sensitivity Analysis of Patients Treated With Metformin Compared With Patients Treated With a Sulfonylurea After Propensity Score Matching **eAppendix.** Statistical Analysis Plan

Supplement 2.
Data Sharing Statement

## References

[1] Centers for Disease Control and Prevention. Osteoarthritis (OA). Accessed February 13, 2023. https://www.cdc.gov/arthritis/basics/osteoarthritis.htm

[2] United States Bone and Joint Initiative. Musculoskeletal diseases and the burden they cause in the United States. Accessed April 22, 2021. https://www.boneandjointburden.org

[3] GBD 2017 Disease and Injury Incidence and Prevalence Collaborators. Global, regional, and national incidence, prevalence, and years lived with disability for 354 diseases and injuries for 195 countries and territories, 1990-2017: a systematic analysis for the Global Burden of Disease Study 2017. Lancet. 2018;392(10159):1789-1858. doi:10.1016/S0140-6736(18)32279-7 30496104 PMC6227754

[4] Hunter DJ, Bierma-Zeinstra S. Osteoarthritis. Lancet. 2019;393(10182):1745-1759. doi:10.1016/S0140-6736(19)30417-9 31034380

[5] Foretz M, Guigas B, Viollet B. Understanding the glucoregulatory mechanisms of metformin in type 2 diabetes mellitus. Nat Rev Endocrinol. 2019;15(10):569-589. doi:10.1038/s41574-019-0242-2 31439934

[6] Flory J, Lipska K. Metformin in 2019. JAMA. 2019;321(19):1926-1927. doi:10.1001/jama.2019.3805 31009043 PMC7552083

[7] Saisho Y. Metformin and inflammation: its potential beyond glucose-lowering effect. Endocr Metab Immune Disord Drug Targets. 2015;15(3):196-205. doi:10.2174/1871530315666150316124019 25772174

[8] Wang Y, Hussain SM, Wluka AE, . Association between metformin use and disease progression in obese people with knee osteoarthritis: data from the Osteoarthritis Initiative-a prospective cohort study. Arthritis Res Ther. 2019;21(1):127. doi:10.1186/s13075-019-1915-x 31126352 PMC6534888

[9] Li J, Zhang B, Liu WX, . Metformin limits osteoarthritis development and progression through activation of AMPK signalling. Ann Rheum Dis. 2020;79(5):635-645. doi:10.1136/annrheumdis-2019-216713 32156705 PMC7213329

[10] Li D, Ruan G, Zhang Y, . Metformin attenuates osteoarthritis by targeting chondrocytes, synovial macrophages and adipocytes. Rheumatology (Oxford). Published online August 19, 2022. doi:10.1093/rheumatology/keac467 35984286

[11] Park MJ, Moon SJ, Baek JA, . Metformin augments anti-inflammatory and chondroprotective properties of mesenchymal stem cells in experimental osteoarthritis. J Immunol. 2019;203(1):127-136. doi:10.4049/jimmunol.1800006 31142603

[12] Li H, Ding X, Terkeltaub R, . Exploration of metformin as novel therapy for osteoarthritis: preventing cartilage degeneration and reducing pain behavior. Arthritis Res Ther. 2020;22(1):34. doi:10.1186/s13075-020-2129-y 32087740 PMC7036179

[13] Lu CH, Chung CH, Lee CH, . Combination COX-2 inhibitor and metformin attenuate rate of joint replacement in osteoarthritis with diabetes: a nationwide, retrospective, matched-cohort study in Taiwan. PLoS One. 2018;13(1):e0191242. doi:10.1371/journal.pone.0191242 29385156 PMC5791980

[14] Chen S, Ruan G, Zeng M, . Association between metformin use and risk of total knee arthroplasty and degree of knee pain in knee osteoarthritis patients with diabetes and/or obesity: a retrospective study. J Clin Med. 2022;11(16):4796. doi:10.3390/jcm11164796 36013035 PMC9409735

[15] Lai FTT, Yip BHK, Hunter DJ, . Metformin use and the risk of total knee replacement among diabetic patients: a propensity-score-matched retrospective cohort study. Sci Rep. 2022;12(1):11571. doi:10.1038/s41598-022-15871-7 35798867 PMC9262887

[16] Kovesdy C, Schmedt N, Folkerts K, . Predictors of cardio-kidney complications and treatment failure in patients with chronic kidney disease and type 2 diabetes treated with SGLT2 inhibitors. BMC Med. 2022;20(1):2. doi:10.1186/s12916-021-02191-2 35000594 PMC8744296

[17] Khokhar B, Jette N, Metcalfe A, . Systematic review of validated case definitions for diabetes in *ICD-9*–coded and *ICD-10*–coded data in adult populations. BMJ Open. 2016;6(8):e009952. doi:10.1136/bmjopen-2015-009952 27496226 PMC4985868

[18] Baker MC, Weng Y, Robinson WH, Ahuja N, Rohatgi N. Reduction in osteoarthritis risk after treatment with ticagrelor compared to clopidogrel: a propensity score-matching analysis. Arthritis Rheumatol. 2020;72(11):1829-1835. doi:10.1002/art.41412 32564514 PMC7722213

[19] Suissa S, Moodie EE, Dell’Aniello S. Prevalent new-user cohort designs for comparative drug effect studies by time-conditional propensity scores. Pharmacoepidemiol Drug Saf. 2017;26(4):459-468. doi:10.1002/pds.4107 27610604

[20] Suissa S, Azoulay L. Metformin and the risk of cancer: time-related biases in observational studies. Diabetes Care. 2012;35(12):2665-2673. doi:10.2337/dc12-0788 23173135 PMC3507580

[21] Allen KD. Racial and ethnic disparities in osteoarthritis phenotypes. Curr Opin Rheumatol. 2010;22(5):528-532. doi:10.1097/BOR.0b013e32833b1b6f 20473172

[22] Quan H, Li B, Couris CM, . Updating and validating the Charlson comorbidity index and score for risk adjustment in hospital discharge abstracts using data from 6 countries. Am J Epidemiol. 2011;173(6):676-682. doi:10.1093/aje/kwq433 21330339

[23] Wasey J. icd. Accessed February 13, 2023. https://github.com/jackwasey/icd

[24] Faries DELA, Haro JM, Obenchain RL. Analysis of Observational Health Care Data Using SAS. SAS Institute; 2010.

[25] Austin PC. Optimal caliper widths for propensity-score matching when estimating differences in means and differences in proportions in observational studies. Pharm Stat. 2011;10(2):150-161. doi:10.1002/pst.433 20925139 PMC3120982

[26] Barnett LA, Jordan KP, Edwards JJ, van der Windt DA. Does metformin protect against osteoarthritis: an electronic health record cohort study. Prim Health Care Res Dev. 2017;18(6):623-628. doi:10.1017/S1463423617000287 28539134

[27] Lim YZ, Wang Y, Estee M, . Metformin as a potential disease-modifying drug in osteoarthritis: a systematic review of pre-clinical and human studies. Osteoarthritis Cartilage. 2022;30(11):1434-1442. doi:10.1016/j.joca.2022.05.005 35597372

[28] Runhaar J, van Middelkoop M, Reijman M, . Prevention of knee osteoarthritis in overweight females: the first preventive randomized controlled trial in osteoarthritis. Am J Med. 2015;128(8):888-895.e4. doi:10.1016/j.amjmed.2015.03.006 25818496

[29] Kostev K, Rex J, Rockel T, Heilmaier C. Effects of selected antidiabetics on weight loss–a retrospective database analysis. Prim Care Diabetes. 2015;9(1):74-77. doi:10.1016/j.pcd.2014.04.001 24815575

[30] Apovian CM, Okemah J, O’Neil PM. Body weight considerations in the management of type 2 diabetes. Adv Ther. 2019;36(1):44-58. doi:10.1007/s12325-018-0824-8 30465123 PMC6318231

