# Supplementary material for: Development of Osteoarthritis in Adults With Type 2 Diabetes Treated With Metformin vs a Sulfonylurea
Source: JAMA Netw Open. 2023 Mar 20;6(3):e233646. doi: 10.1001/jamanetworkopen.2023.3646 (PMC10028483; doi:10.1001/jamanetworkopen.2023.3646)
Supplement: Supplement 1. — eTable 1. Codes Used for Patient Inclusion and Exclusion and Outcome Ascertainment eTable 2. Hazard Ratios of Developing Osteoarthritis and Undergoing Joint Replacement in Patients Treated With Metformin vs a Sulfonylurea, Stratified by Prior Metformin Use in the Sulfonylurea Group eFigure 1. Primary Analysis and Sensitivity Analysis Study Design eFigure 2. Prevalent New-User Study Design With Time-Based Exposure Sets Defined by a 15-Day Interval Surrounding the Timing of Sulfonylurea Initiation eFigure 3. Flowchart of Cohort Selection for Primary Analysis eFigure 4. Flowchart of Cohort Selection for Sensitivity Analysis eFigure 5. Kaplan-Meier Curve of Time to Osteoarthritis Diagnosis in the Sensitivity Analysis of Patients Treated With Metformin Compared With Patients Treated With a Sulfonylurea After Propensity Score Matching eAppendix. Statistical Analysis Plan [file jamanetwopen-e233646-s001.pdf]

Supplemental Online Content

Baker MC, Sheth K, Liu Y, Lu D, Lu R, Robinson WH. Development of osteoarthritis in adults with type 2 diabetes treated with metformin vs a sulfonylurea. *JAMA Netw Open*. 2023;6(3):e233646. doi:10.1001/jamanetworkopen.2023.3646

eTable 1. Codes Used for Patient Inclusion and Exclusion and Outcome Ascertainment
eTable 2. Hazard Ratios of Developing Osteoarthritis and Undergoing Joint Replacement in Patients Treated With Metformin vs a Sulfonylurea, Stratified by Prior Metformin Use in the Sulfonylurea Group
eFigure 1. Primary Analysis and Sensitivity Analysis Study Design
eFigure 2. Prevalent New-User Study Design With Time-Based Exposure Sets Defined by a 15-Day Interval Surrounding the Timing of Sulfonylurea Initiation
eFigure 3. Flowchart of Cohort Selection for Primary Analysis
eFigure 4. Flowchart of Cohort Selection for Sensitivity Analysis
eFigure 5. Kaplan-Meier Curve of Time to Osteoarthritis Diagnosis in the Sensitivity Analysis of Patients Treated With Metformin Compared With Patients Treated With a Sulfonylurea After Propensity Score Matching
eAppendix. Statistical Analysis Plan

This supplemental material has been provided by the authors to give readers additional information about their work.

**eTable 1.** Codes used for patient inclusion and exclusion and outcome ascertainment.	
**ICD Codes for Type 2 Diabetes**	
**ICD-9**	**Diagnosis**
249.00	Secondary diabetes mellitus without mention of complication, not stated as uncontrolled, or unspecified
249.01	Secondary diabetes mellitus without mention of complication, uncontrolled
249.10	Secondary diabetes mellitus with ketoacidosis, not stated as uncontrolled, or unspecified
249.11	Secondary diabetes mellitus with ketoacidosis, uncontrolled
249.20	Secondary diabetes mellitus with hyperosmolarity, not stated as uncontrolled, or unspecified
249.21	Secondary diabetes mellitus with hyperosmolarity, uncontrolled
249.30	Secondary diabetes mellitus with other coma, not stated as uncontrolled, or unspecified
249.31	Secondary diabetes mellitus with other coma, uncontrolled
249.40	Secondary diabetes mellitus with renal manifestations, not stated as uncontrolled, or unspecified
249.41	Secondary diabetes mellitus with renal manifestations, uncontrolled
249.50	Secondary diabetes mellitus with ophthalmic manifestations, not stated as uncontrolled, or unspecified
249.51	Secondary diabetes mellitus with ophthalmic manifestations, uncontrolled
249.60	Secondary diabetes mellitus with neurological manifestations, not stated as uncontrolled, or unspecified
249.61	Secondary diabetes mellitus with neurological manifestations, uncontrolled
249.70	Secondary diabetes mellitus with peripheral circulatory disorders, not stated as uncontrolled, or unspecified
249.71	Secondary diabetes mellitus with peripheral circulatory disorders, uncontrolled
249.80	Secondary diabetes mellitus with other specified manifestations, not stated as uncontrolled, or unspecified
249.81	Secondary diabetes mellitus with other specified manifestations, uncontrolled
249.90	Secondary diabetes mellitus with unspecified complication, not stated as uncontrolled, or unspecified
249.91	Secondary diabetes mellitus with unspecified complication, uncontrolled
250.00	Diabetes mellitus without mention of complication, type ii or unspecified type, not stated as uncontrolled
250.02	Diabetes mellitus without mention of complication, type ii or unspecified type, uncontrolled

© 2023 Baker MC et al. *JAMA Network Open.*

250.10	Diabetes with ketoacidosis, type II or unspecified type, not stated as uncontrolled
250.12	Diabetes with ketoacidosis, type II or unspecified type, uncontrolled
250.20	Diabetes with hyperosmolarity, type II or unspecified type, not stated as uncontrolled
250.22	Diabetes with hyperosmolarity, type II or unspecified type, uncontrolled
250.30	Diabetes with other coma, type II or unspecified type, not stated as uncontrolled
250.32	Diabetes with other coma, type II or unspecified type, uncontrolled
250.40	Diabetes with renal manifestations, type ii or unspecified type, not stated as uncontrolled
250.42	Diabetes with renal manifestations, type II or unspecified type, uncontrolled
250.50	Diabetes with ophthalmic manifestations, type II or unspecified type, not stated as uncontrolled
250.52	Diabetes with ophthalmic manifestations, type II or unspecified type, uncontrolled
250.60	Diabetes with neurological manifestations, type ii or unspecified type, not stated as uncontrolled
250.62	Diabetes with neurological manifestations, type II or unspecified type, uncontrolled
250.70	Diabetes with peripheral circulatory disorders, type ii or unspecified type, not stated as uncontrolled
250.72	Diabetes with peripheral circulatory disorders, type II or unspecified type, uncontrolled
250.80	Diabetes with other specified manifestations, type ii or unspecified type, not stated as uncontrolled
250.82	Diabetes with other specified manifestations, type II or unspecified type, uncontrolled
250.90	Type 2 diabetes mellitus with unspecified complications
250.92	Diabetes with unspecified complication, type II or unspecified type, uncontrolled
**ICD-10**	**Diagnosis**
E08.00, E08.01, E08.10, E08.11, E08.21, E08.22, E08.29, E08.311, E08.319, E08.3211, E08.3212, E08.3213, E08.3219, E08.3291, E08.3292, E08.3293, E08.3299, E08.3311, E08.3312, E08.3313, E08.3319, E08.3391, E08.3392, E08.3393, E08.3399, E08.3411, E08.3412, E08.3413, E08.3419, E08.3491, E08.3492, E08.3493, E08.3499, E08.3511, E08.3512, E08.3513, E08.3519, E08.3521, E08.3522, E08.3523, E08.3529, E08.3531, E08.3532, E08.3533, E08.3539, E08.3541, E08.3542, E08.3543, E08.3549, E08.3551, E08.3552, E08.3553, E08.3559, E08.3591, E08.3592, E08.3593, E08.3599, E08.36, E08.37X1, E08.37X2, E08.37X3, E08.37X9, E08.39, E08.40, E08.41, E08.42, E08.43, E08.44, E08.49, E08.51, E08.52, E08.59, E08.610, E08.618, E08.620, E08.621, E08.622, E08.628, E08.630, E08.638, E08.641, E08.649, E08.65, E08.69, E08.8, E08.9	Diabetes mellitus due to underlying condition
E09.00, E09.01, E09.10, E09.11, E09.21, E09.22, E09.29, E09.311, E09.319, E09.3211, E09.3212, E09.3213, E09.3219, E09.3291, E09.3292, E09.3293, E09.3299, E09.3311, E09.3312, E09.3313, E09.3319, E09.3391, E09.3392, E09.3393, E09.3399, E09.3411, E09.3412, E09.3413, E09.3419, E09.3491, E09.3492, E09.3493, E09.3499, E09.3511, E09.3512, E09.3513, E09.3519, E09.3521, E09.3522, E09.3523, E09.3529, E09.3531, E09.3532, E09.3533, E09.3539, E09.3541, E09.3542, E09.3543, E09.3549,	Drug or chemical induced diabetes mellitus

© 2023 Baker MC et al. *JAMA Network Open.*

E09.3551, E09.3552, E09.3553, E09.3559, E09.3591, E09.3592, E09.3593, E09.3599, E09.36, E09.37X1, E09.37X2, E09.37X3, E09.37X9, E09.39, E09.40, E09.41, E09.42, E09.43, E09.44, E09.49, E09.51, E09.52, E09.59, E09.610, E09.618, E09.620, E09.621, E09.622, E09.628, E09.630, E09.638, E09.641, E09.649, E09.65, E09.69, E09.8 E09.9	
E11.00, E11.01, E11.10, E11.11, E11.21, E11.22, E11.29, E11.311, E11.319, E11.3211, E11.3212, E11.3213, E11.3219, E11.3291, E11.3292, E11.3293, E11.3299, E11.3311, E11.3312, E11.3313, E11.3319, E11.3391, E11.3392, E11.3393, E11.3399, E11.3411, 11.3412, E11.3413, E11.3419, E11.3491, E11.3492, E11.3493, E11.3499, E11.3511, E11.3512, E11.3513, E11.3519, E11.3521, E11.3522, E11.3523, E11.3529, E11.3531, E11.3532, E11.3533, E11.3539, E11.3541, E11.3542, E11.3543, E11.3549, E11.3551, E11.3552, E11.3553, E11.3559, E11.3591, E11.3592, E11.3593, E11.3599, E11.36, E11.37X1, E11.37X2, E11.37X3, E11.37X9, E11.39, E11.40, E11.41, E11.42, E11.43, E11.44, E11.49, E11.51, E11.52, E11.59, E11.610, E11.618, E11.620, E11.621, E11.622, E11.628, E11.630, E11.638, E11.641, E11.649, E11.65, E11.69, E11.8, E11.9	Type 2 diabetes mellitus
E13.00, E13.01, E13.10, E13.11, E13.21, E13.22, E13.29, E13.311, E13.319, E13.3211, E13.3212, E13.3213, E13.3219, E13.3291, E13.3292, E13.3293, E13.3299, E13.3311, E13.3312, E13.3313, E13.3319, E13.3391, E13.3392, E13.3393, E13.3399, E13.3411, E13.3412, E13.3413, E13.3419, E13.3491, E13.3492, E13.3493, E13.3499, E13.3511, E13.3512, E13.3513, E13.3519, E13.3521, E13.3522, E13.3523, E13.3529, E13.3531, E13.3532, E13.3533, E13.3539, E13.3541, E13.3542, E13.3543, E13.3549, E13.3551, E13.3552, E13.3553, E13.3559, E13.3591, E13.3592, E13.3593, E13.3599, E13.36, E13.37X1, E13.37X2, E13.37X3, E13.37X9, E13.39, E13.40, E13.41, E13.42, E13.43, E13.44, E13.49, E13.51, E13.52, E13.59, E13.610, E13.618, E13.620, E13.621, E13.622, E13.628, E13.630, E13.638, E13.641, E13.649, E13.65, E13.69, E13.8, E13.9	Other specified diabetes mellitus
**ICD Codes for Osteoarthritis**	
**ICD-9**	**Diagnosis**
715	Osteoarthritis and allied disorders
715.90	Osteoarthrosis unspecified
715.09	Osteoarthrosis multiple sites
715.00	Osteoarthrosis site unspecified
715.98	Osteoarthrosis other sites
715.89	Osteoarthrosis multiple sites
715.80	Osteoarthrosis unspecified
715.18	Osteoarthrosis other sites
715.10	Osteoarthrosis unspecified
715.30	Osteoarthrosis unspecified
715.38	Osteoarthrosis other sites
715.9	Osteoarthrosis generalized or localized
715.1	Osteoarthrosis localized primary
715.0	Osteoarthrosis generalized
715.20	Osteoarthrosis site unspecified
715.28	Osteoarthrosis and other sites

© 2023 Baker MC et al. *JAMA Network Open.*

715.3	Osteoarthrosis not specified
715.8	Osteoarthrosis not specified
715.16	Osteoarthrosis, localized, primary, lower leg
715.26	Osteoarthrosis, localized, secondary, lower leg
715.36	Osteoarthrosis, localized, not specified whether primary or secondary, lower leg
715.96	Osteoarthrosis, unspecified whether generalized or localized, lower leg
715.15	Osteoarthrosis, localized, primary, pelvic region and thigh
715.25	Osteoarthrosis, localized, secondary, pelvic region and thigh
715.35	Osteoarthrosis, localized, not specified whether primary or secondary, pelvic region and thigh
715.95	Osteoarthrosis, unspecified whether generalized or localized, pelvic region and thigh
715.04	Osteoarthrosis, hand
715.14	Osteoarthrosis, localized, primary, hand
715.24	Osteoarthrosis, localized, secondary, hand
715.34	Osteoarthrosis, localized, not specified whether primary or secondary, hand
715.94	Osteoarthrosis, unspecified whether generalized or localized, hand
**ICD-10**	**Diagnosis**
M15	Polyosteoarthritis
M16	Osteoarthritis of hip
M17	Osteoarthritis of knee
M18	Osteoarthritis of first carpometacarpal joint
M19	Other and unspecified osteoarthritis
M19.90	Osteoarthritis unspecified
M15.0	Primary generalized osteoarthritis
M15.9	Polyosteoarthritis unspecified
M19.91	Primary osteoarthritis unspecified
M15.8	Other polyosteoarthritis
M19.93	Osteoarthritis unspecified site
M19.92	Osteoarthritis unspecified site
M17.11	Osteoarthritis right knee
M17.12	Osteoarthritis left knee
M17.0	Osteoarthritis bilateral knee
M17.9	Osteoarthritis knee unspecified
M17.31	Osteoarthritis right knee
M17.32	Osteoarthritis left knee
M17.5	Osteoarthritis knee
M17.2	Osteoarthritis bilateral knee
M17.4	Osteoarthritis bilateral knee
M17.30	Osteoarthritis unspecified knee

© 2023 Baker MC et al. *JAMA Network Open.*

M16.11	Osteoarthritis right hip
M16.12	Osteoarthritis left hip
M16.0	Osteoarthritis bilateral hip
M16.9	Osteoarthritis unspecified hip
M16.10	Osteoarthritis unspecified hip
M16.7	Osteoarthritis hip
M16.51	Osteoarthritis right hip
M16.52	Osteoarthritis left hip
M16.31	Osteoarthritis right hip
M16.32	Osteoarthritis left hip
M16.2	Osteoarthritis bilateral hip
M166	Osteoarthritis bilateral hip
M16.4	Osteoarthritis bilateral hip
M19.04	Primary osteoarthritis, hand
M19.14	Post-traumatic osteoarthritis, hand
M19.24	Secondary osteoarthritis, hand
M19.041	Osteoarthritis right hand
M19.042	Osteoarthritis left hand
M18.12	Osteoarthritis first CMC left hand
M18.11	Osteoarthritis first CMC right hand
M19.049	Osteoarthritis unspecified hand
M18.0	Osteoarthritis bilateral first CMC joints
M18.9	Osteoarthritis first CMC joint unspecified
M15.1	Heberden’s nodes with arthropathy
M15.2	Bouchard’s nodes with arthropathy
M18.10	Osteoarthritis unspecified hand
M19.241	Osteoarthritis right hand
M19.141	Osteoarthritis right hand
M19.242	Osteoarthritis left hand
M18.2	Osteoarthritis bilateral first CMC joints
M18.4	Osteoarthritis other bilateral first CMC joints
M18.30	Osteoarthritis first CMC joint unspecified hand
M19.142	Osteoarthritis left hand
M18.32	Osteoarthritis first CMC left hand
M18.52	Osteoarthritis first CMC left hand
M18.31	Osteoarthritis first CMC right hand
M18.51	Osteoarthritis first CMC right hand
M19.249	Osteoarthritis unspecified hand
**ICD Codes for Inflammatory Arthritis**	
**ICD-9**	**Diagnosis**
710.0	Systemic lupus erythematosus

© 2023 Baker MC et al. *JAMA Network Open.*

714.0, 714.1, 714.2, 714.81	Rheumatoid arthritis
696.0	Psoriatic arthritis
710.2	Sjogren's syndrome
720.0, 720.1, 720.2, 720.81, 720.89, 720.9	Ankylosing spondylitis
714.30, 714.31, 714.32, 714.33, 714.81	Juvenile rheumatoid arthritis
714.89, 714.9, 714.90	Inflammatory polyarthropathy
714.2	Adult-onset Still's disease
733.4	Osteonecrosis
**ICD-10**	**Diagnosis**
M32.1, M32.10, M32.14, M32.19, M32.8, M32.9	Systemic lupus erythematosus
M06.00, M06.011, M06.012, M06.019, M06.021, M06.022, M06.029, M06.031, M06.032, M06.039, M06.041, M06.042, M06.049, M06.051, M06.052, M06.059, M06.061, M06.062, M06.069, M06.071, M06.072, M06.079, M06.08, M06.09, M06.0A, M06.811, M06.812, M06.819, M06.821, M06.822, M06.829, M06.831, M06.832, M06.839, M06.841, M06.842, M06.849, M06.851, M06.852, M06.859, M06.861, M06.862, M06.869, M06.871, M06.872, M06.879, M06.88, M06.8, M06.8A, M06.9, M05.60, M05.631, M05.632, M05.639, M05.641, M05.642, M05.649, M05.651, M05.652, M05.659, M05.661, M05.662, M05.669, M05.671, M05.672, M05.679, M05.69, M05.70, M05.711, M05.712, M05.719, M05.721, M05.722, M05.729, M05.731, M05.732, M05.739, M05.741, M05.742, M05.749, M05.751, M05.752, M05.759, M05.761, M05.762, M05.769, M05.771, M05.772, M05.779, M05.79, M05.7A, M05.80, M05.811, M05.812, M05.819, M05.821, M05.822, M05.829, M05.831, M05.832, M05.839, M05.841, M05.842, M05.849, M05.851, M05.852, M05.859, M05.861, M05.862, M05.869, M05.871, M05.872, M05.879, M05.89, M05.8A, M05.9	Rheumatoid arthritis
L40.50, L40.51, L40.52, L40.53, L40.54, L40.59	Psoriatic arthritis
M35.00, M35.01, M35.02, M35.03, M35.04, M35.09	Sjogren's syndrome
M45.0, M45.1, M45.2, M45.3, M45.4, M45.5, M45.6, M45.7, M45.8, M45.9, M46.90	Ankylosing spondylitis
M08.09, M08.0, M08.20, M08.29	Juvenile rheumatoid arthritis
M06.4	Inflammatory polyarthropathy
M06.1	Adult-onset Still's disease
M87	Osteonecrosis
**CPT Codes for Arthroplasty**	
**CPT Code**	**Diagnosis**
27120, 27122, 27125, 27130	Hip arthroplasty
27438, 27440, 27441, 27442, 27443, 27445, 27446, 27447	Knee arthroplasty

CPT = current procedural terminology; ICD = international classification of diseases.

© 2023 Baker MC et al. *JAMA Network Open.*

**eTable 2.** Hazard ratios of developing osteoarthritis and undergoing joint replacement in patients treated with metformin versus a sulfonylurea stratified by prior metformin usage in the sulfonylurea group.		
	**Metformin Patients Compared with Sulfonylurea Patients with Prior Metformin Use (*n* = 6876 Pairs)**	**Metformin Patients Compared with Sulfonylurea Patients with No Prior Metformin Use (*n* = 14061 Pairs)**
**Incident OA**		
Crude HR (95% CI)	0.81 (0.67-0.98)	0.64 (0.56-0.73)
Adjusted HR (95% CI)*	0.92 (0.76-1.12)	0.71 (0.62-0.81)
**Joint replacement**		
Crude HR (95% CI)	0.72 (0.34-1.53)	0.69 (0.38-1.24)
Adjusted HR (95% CI)*	0.84 (0.39-1.81)	0.76 (0.42-1.40)

OA = osteoarthritis; HR = hazard ratio; 95% CI = 95% confidence interval. *Adjusted for age, sex race, geographical region, education, Charlson comorbidity score, and outpatient visit frequency.

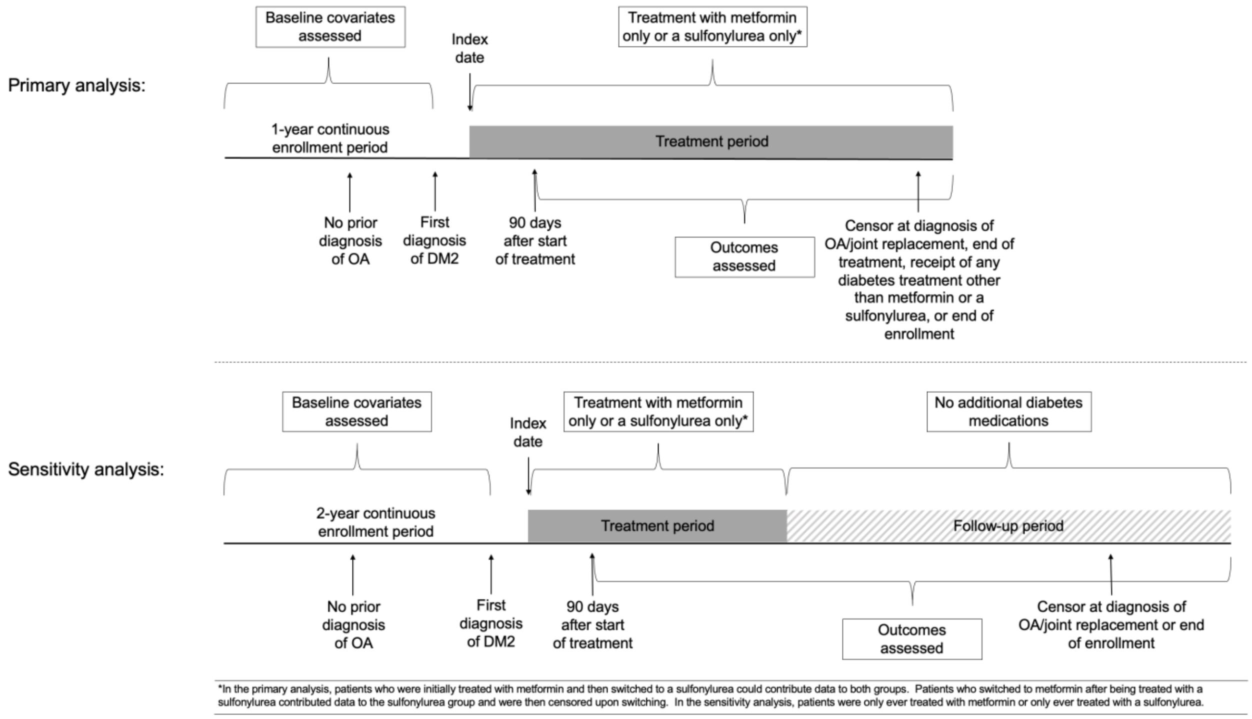

eFigure 1. Primary analysis and sensitivity analysis study design.

© 2023 Baker MC et al. *JAMA Network Open.*

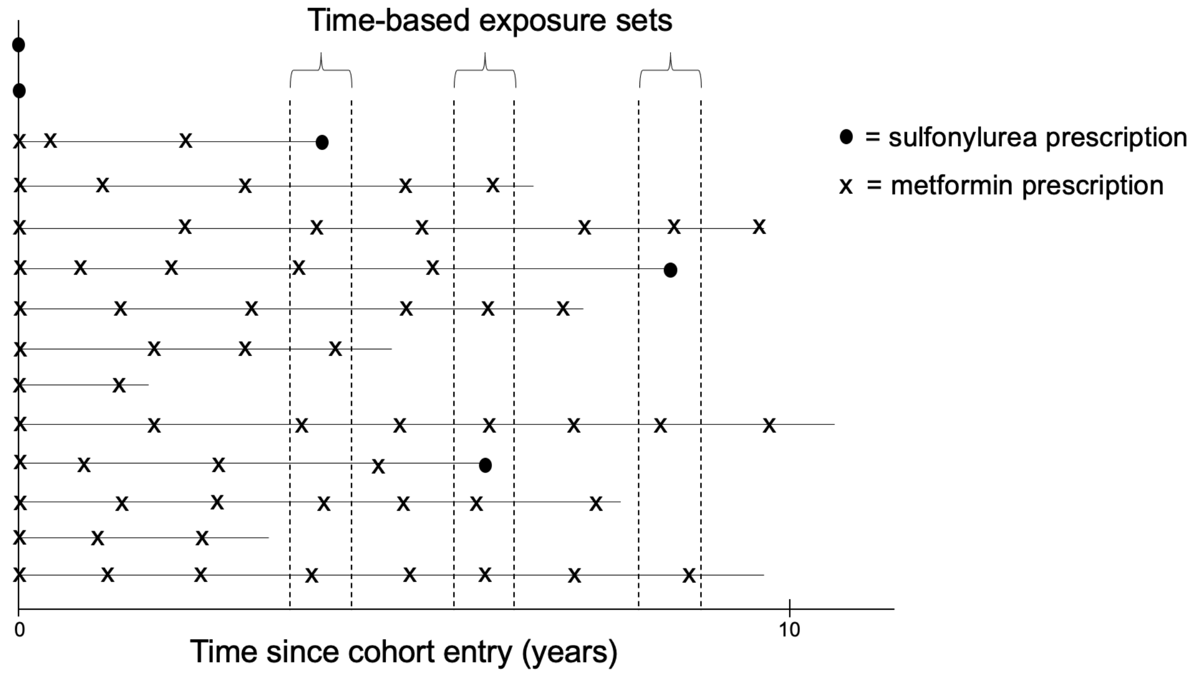

eFigure 2. Prevalent new-user study design with time-based exposure sets defined by a 15-day interval surrounding the timing of sulfonylurea initiation.

Figure adopted from: Suissa S, Moodie EE, Dell'Aniello S. Prevalent new-user cohort designs for comparative drug effect studies by time-conditional propensity scores. Pharmacoepidemiol Drug Saf. 2017 Apr; 26(4):459-468.

© 2023 Baker MC et al. *JAMA Network Open.*

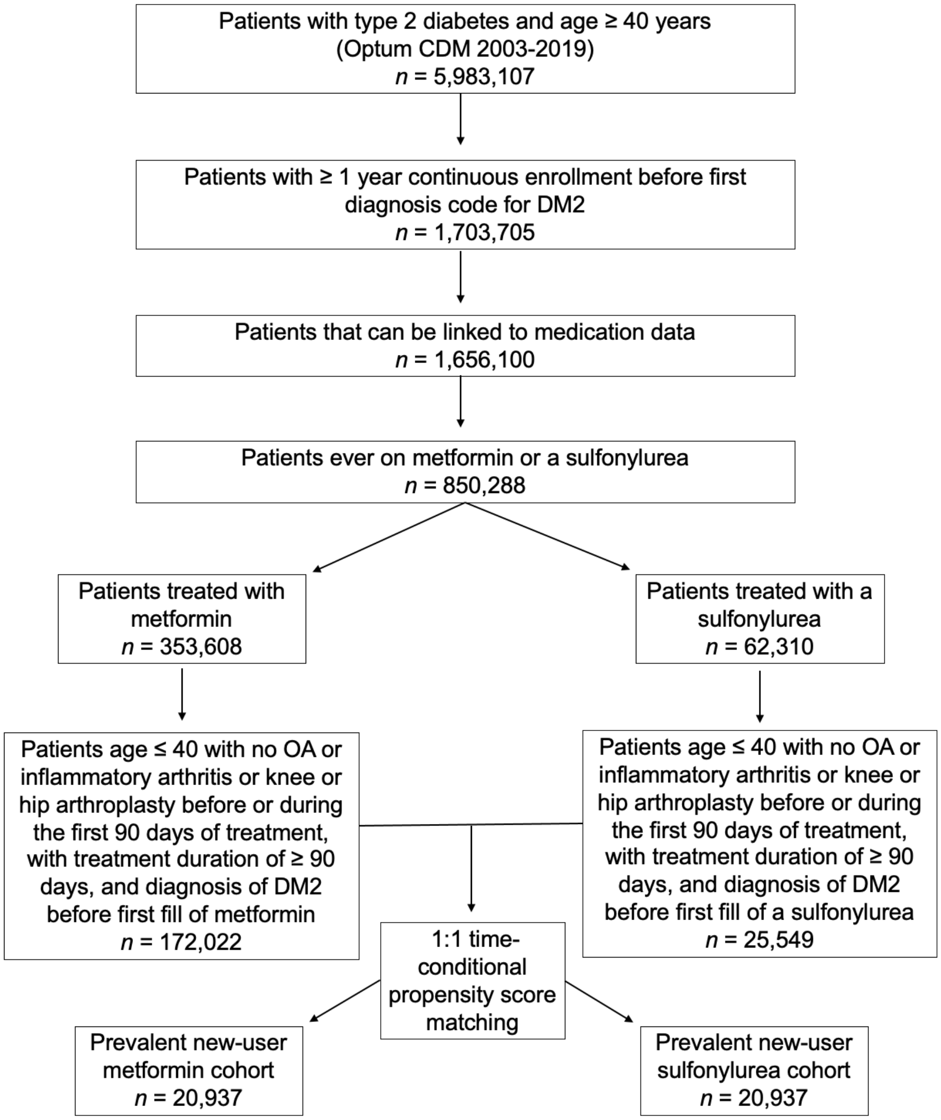

eFigure 3: Flow chart of cohort selection for primary analysis.

© 2023 Baker MC et al. *JAMA Network Open.*

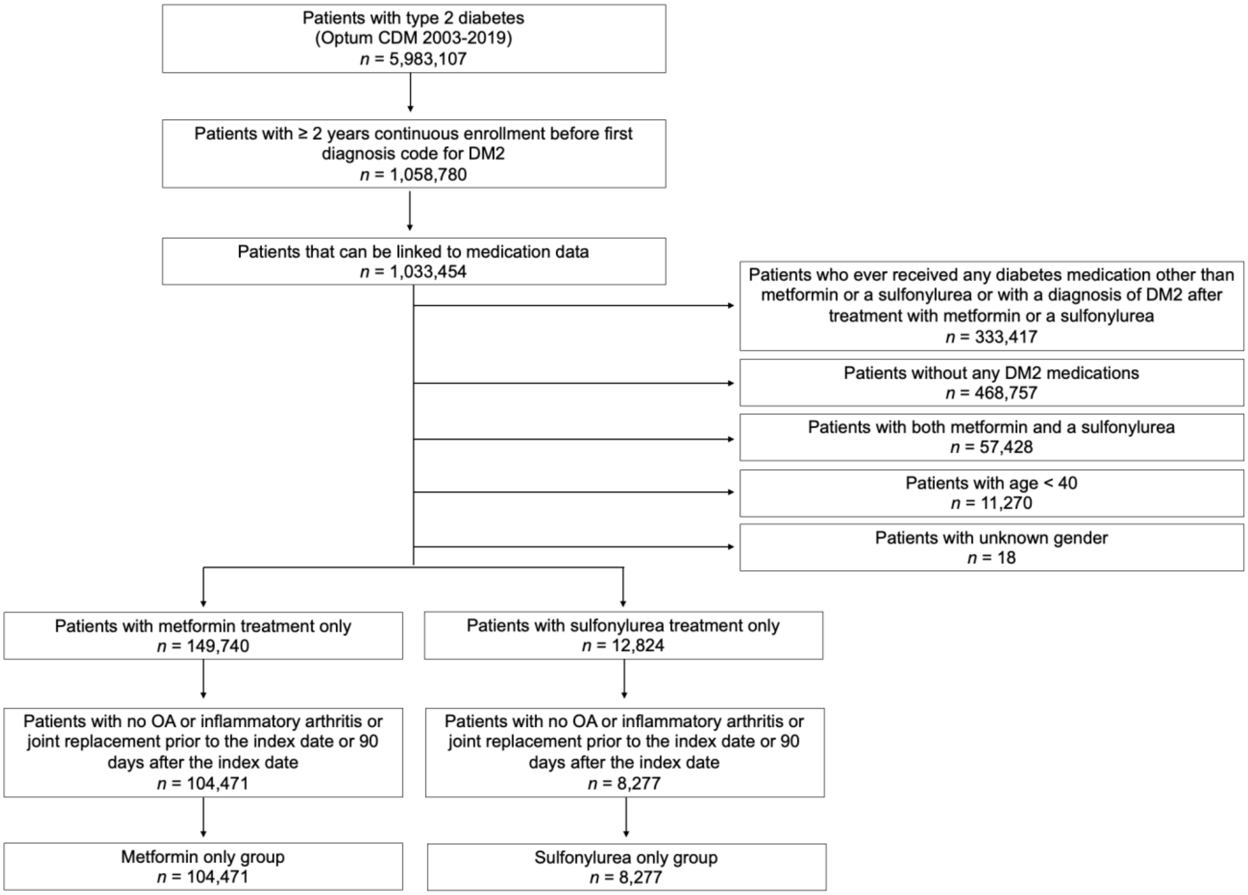

eFigure 4: Flow chart of cohort selection for sensitivity analysis.

© 2023 Baker MC et al. *JAMA Network Open.*

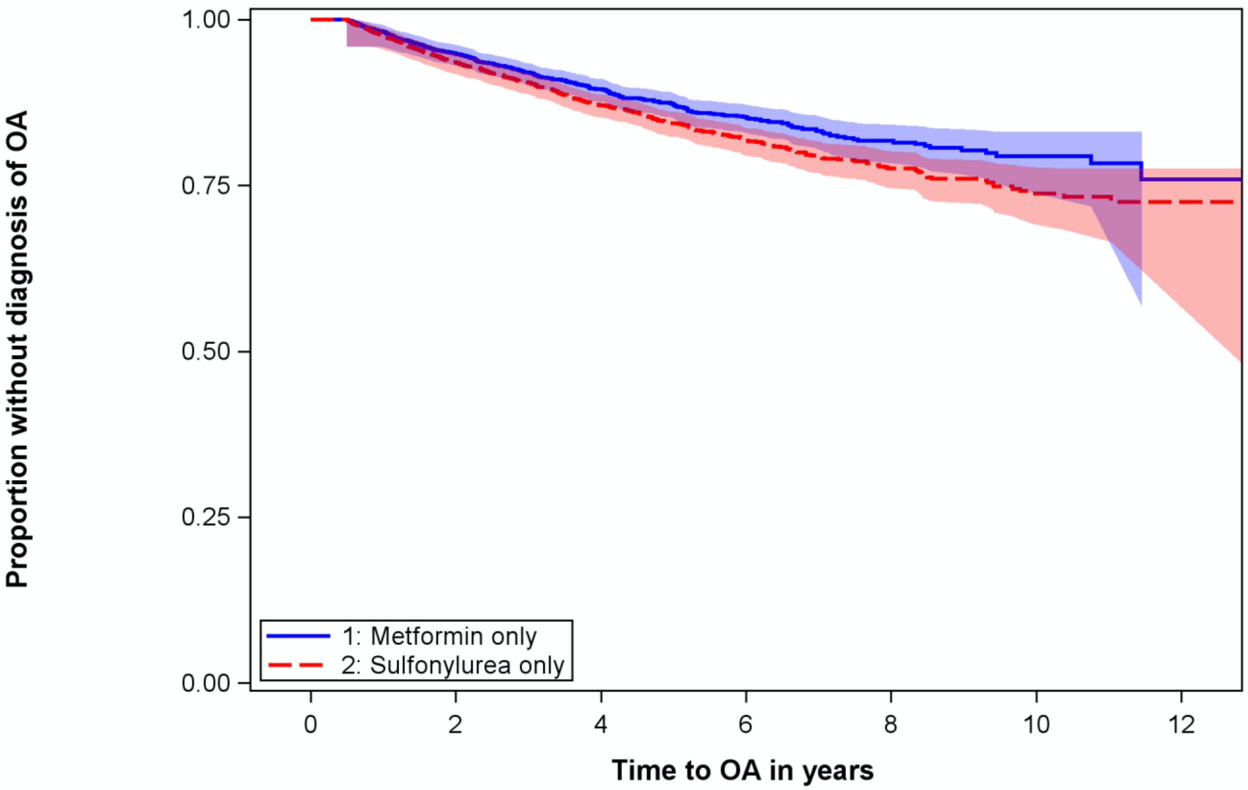

Number at risk

	0	2	4	6	8	10	12
1: Metformin only	8277	3821	1605	710	344	123	21
2: Sulfonylurea only	8277	3905	1918	959	475	185	47

eFigure 5: Kaplan-Meier curve of time to osteoarthritis diagnosis in the sensitivity analysis of metformin treated patients compared with sulfonylurea treated patients after propensity score matching.

© 2023 Baker MC et al. *JAMA Network Open.*

eText 1: Statistical analysis plan.

1. Study Overview

1.1 Study Aims

To determine the risk of osteoarthritis (OA) and joint replacement in individuals with type 2 diabetes mellitus (DM2) treated with metformin compared with a sulfonylurea.

1.2 Primary Hypothesis
Patients treated with metformin will have a lower incidence of osteoarthritis compared with patients treated with a sulfonylurea.

2. Study Population

Inclusion criteria:

- Individuals 40 years of age or older
- Individuals with at least one year of continuous enrollment in the Optum CDM database before the first International Classification of Diseases (ICD)-9 or ICD-10 diagnosis for DM2
- Patients with DM2, defined as having at least two ICD-9/10 codes for DM2 separated by 14 days or more (eTable 1)

Exclusion criteria:

- Individuals with the first diagnosis of diabetes occurring after the start date of metformin or the sulfonylurea
- Individuals with type 1 diabetes (DM1)
- Individuals with prior diagnoses of OA, any inflammatory arthritis, or with joint replacement based on Current Procedural Terminology (CPT) codes, prior to the treatment start date or within the first 90 days of the treatment start date (eTable 1)
- Individuals started on metformin and a sulfonylurea at the same time
- Individuals on combination metformin and/or sulfonylurea medications

3. Outcomes, Exposures, and Additional Variables for Interest

3.1 Primary Outcome(s)

- Any OA: two ICDs codes ever, separated by 14 days or more. The date of the event will be defined as the date of the first of the two ICD codes.
- Joint replacement: one CPT code for joint replacement.

3.2 Exposures

Treatment episode definitions:

© 2023 Baker MC et al. *JAMA Network Open.*

- For individuals who only received metformin or a sulfonylurea: No prescription for longer than 90 days was considered as a gap. We separated treatment episodes by gap and only included treatment episodes greater than 90 days (multiple episodes could be included for one patient if the length was greater than 90 days)

- For patients who switched between the two medications:
    - For individuals who switched from metformin to a sulfonylurea (with or without later switch): Treatment after the second switch was not included. No prescription for longer than 90 days was considered as a gap. We separated treatment episodes by gap and only included treatment episodes greater than 90 days (these patients could be included in both metformin and sulfonylurea cohorts)

    - For individuals who switched from a sulfonylurea to metformin (with or without later switch): Treatment after the first switch was not included (only the sulfonylurea episode was included). We separated treatment episodes by gap and only included treatment episodes greater than 90 days (these patients were only included in the sulfonylurea cohort)

Treatment censored at the time:

- Receiving any of below meds: Insulin, repaglinide, Nateglinide, pioglitazone, Rosiglitazone, Albiglutide, Dulaglutide, Exenatide, Liraglutide, Lixisenatide, Semaglutide, empagliflozin, canagliflozin, dapagliflozin, Ertugliflozin, Alogliptin, Linagliptin, Saxagliptin, Sitagliptin
- Developing the outcome of interest
- No longer present in the Optum CDM database
- End of the follow-up period (December 31, 2019)

3.3 Additional Variables of Interest

Variable name	Description	Type
Age	Age at first diagnosis of diabetes	Continuous
Gender	Gender	Categorical
Race	Race	Categorical
Region	Census level Division based on US State. If one patient has multiple regions recorded in system, use the region recorded closet to first diagnosis of diabetes.	Categorical
Education	Education level	Categorical
Charlson comorbidity score	Quan-Deyo method will be used for calculation using diagnosis record from one year before to first diagnosis of diabetes. Categorized into 5 groups due to the skewed distribution to be included in analysis.	Continuous

© 2023 Baker MC et al. *JAMA Network Open.*

Yearly outpatient visit	Yearly outpatient visit number during follow-up period	Continuous
Follow-up time	Time from 90 days after treatment period start until switching to other treatment, developing an event, no longer present in the Optum CDM database, or until the end of the follow-up period (December 31, 2019).	Continuous

4. Statistical Analysis Plan

4.1 Demographic and Clinical Characteristics

Demographic and clinical variables were extracted and compared by cohort. The results are presented in Table 1.

Descriptive statistics are presented as frequencies and percentages for categorical variables. Continuous variables are presented as means with standard deviation (SD) and means with interquartile ranges (IQR).

4.2 Analysis Plan for Primary hypothesis

The primary analysis utilizing a prevalent new-user cohort design was conducted comparing the first-line treatment (metformin) with the second-line treatment (sulfonylurea) using time-based exposure sets to identify matched subjects at the same point in the course of disease (eFigure 2). The cohort included all individuals treated with a sulfonylurea. For each person treated with a sulfonylurea, a matched person treated with metformin was identified based on time-based exposure sets defined as time intervals (+/- 15 days) from the first prescription of metformin to the first dose of sulfonylurea. Individuals were matched 1:1 on time-conditional propensity scores using conditional logistic regression adjusting for age, sex, race, Charlson comorbidity score (categorized), and treatment duration to estimate the propensity to receive a sulfonylurea. For time-conditional propensity score matching, we started chronologically with the first individual prescribed a sulfonylurea and selected the individual from the exposure set with the closest time-conditional propensity score. Once a person was selected into the comparator group, they were no longer considered in subsequent exposure sets as potential comparators.

Incidence rates (IRs) and the 95% Wald confidence intervals (95% CIs) were calculated for developing OA and undergoing joint replacement and Cox proportional hazard models were used to assess the hazard ratio (HR) and 95% CI of developing OA and joint replacement among individuals with DM2 treated with metformin compared with a sulfonylurea after adjusting for age, sex race, geographical region, education, Charlson comorbidity score, and outpatient visit frequency. IRs are reported as the number of events per 1,000 person years. Kaplan-Meier curves were created to report the probability of developing OA over a certain time interval. A stratified analysis by individuals treated with a sulfonylurea with prior metformin exposure or without prior

© 2023 Baker MC et al. *JAMA Network Open.*

metformin exposure was also conducted using the matched pairs. This evaluated the treatment effect of metformin compared with a sulfonylurea.

5. Sensitivity Analysis:

The robustness of our results was examined through a sensitivity analysis comparing metformin-treated patients with sulfonylurea-treated patients that were only ever treated with those medications, allowing for lifetime follow-up of the outcome (even after patients had stopped the medication of interest). We included patients aged 40 years or older with more than two years of continuous enrollment in the Optum database before the first ICD-9 or ICD-10 diagnosis of DM2. For the metformin group, we included DM2 patients who were ever treated with metformin and never treated with any additional diabetes medications during their entire follow-up period. For the sulfonylurea group, we included DM2 patients who were ever treated with a sulfonylurea medication and never treated with any additional diabetes medications during their entire follow-up period. We excluded patients with type I DM, patients who ever received any diabetes medication other than metformin including combination metformin drugs, and patients with the first diagnosis of diabetes occurring after the start date of metformin. Patients with prior diagnoses of OA or any inflammatory arthritis prior to the index date or within the first 90 days of the index date were also excluded. Similarly, patients with joint replacement based on CPT codes prior to the index date or within the first 90 days of the index date were excluded. For both groups, the index date was the first fill date for the drug of interest. Patients were followed from the 90 days after the index date until they were newly diagnosed with OA, underwent knee or hip joint arthroplasty, were no longer present in the Optum database, or until the end of the follow-up period (December 31, 2019) (Supplementary Figure 1). The primary endpoint was the time to diagnosis of incident OA starting 90 days after the index date, defined as two or more ICD-9 or ICD-10 codes for OA separated by 14 days or more. The secondary endpoint was the time to joint replacement starting 90 days after the index date, defined as a documented CPT code for hip or knee joint replacement. We conducted 1:1 propensity score matching (individuals treated with metformin versus a sulfonylurea) using age, sex, race, Charlson comorbidity score and treatment duration. Patient characteristics were compared before and after propensity score matching. IRs and the 95% CIs were calculated for developing OA and undergoing joint replacement and Cox proportional hazard models were used to assess the HR and 95% CI of developing OA and joint replacement among DM2 patients treated with metformin compared with a sulfonylurea after adjusting for age, sex race, Charlson comorbidity score, and treatment duration. Kaplan-Meier curves were created after propensity score matching.

The analysis was done with SAS using %gmatch macro and R 4.1.1 using below packages:
icd: calculate Charlson comorbidity scores
tableone: calculate standardized mean differences (SMD)
survival: Cox-PH model
exactci: calculate confidence interval for incidence rate per patient year
fmsb: calculate incidence rate ratio

© 2023 Baker MC et al. *JAMA Network Open.*
