# Supplementary material for: Development of Osteoarthritis in Adults With Type 2 Diabetes Treated With Metformin vs a Sulfonylurea
Source: JAMA Netw Open. 2023 Mar 20;6(3):e233646. doi: 10.1001/jamanetworkopen.2023.3646 (PMC10028483; doi:10.1001/jamanetworkopen.2023.3646)
Supplement: Supplement 2. — Data Sharing Statement [file jamanetwopen-e233646-s002.pdf]

Data Sharing Statement

Baker. Development of Osteoarthritis in Adults With Type 2 Diabetes Treated With Metformin vs a Sulfonylurea. *JAMA Netw Open*. Published March 20, 2023.
doi:10.1001/jamanetworkopen.2023.3646

Data
Data available: No
